# Red yeast rice repairs kidney damage and reduces inflammatory transcription factors in rat models of hyperlipidemia

**DOI:** 10.3892/etm.2014.2035

**Published:** 2014-10-21

**Authors:** MEI DING, DAOYUAN SI, WENQI ZHANG, ZHAOHUI FENG, MIN HE, PING YANG

**Affiliations:** Department of Cardiology, China-Japan Union Hospital of Jilin University, Changchun, Jilin 130033, P.R. China

**Keywords:** xuezhikang, hyperlipidimia, kidney function, tumor necrosis factor-α, interleukin-6

## Abstract

Xuezhikang (XZK), an extract of red yeast rice, has been widely used for the management of hyperlipidemia and coronary heart disease (CHD); however, the effects of XZK treatment on kidney injury have not yet been fully identified. The aim of the current study was to evaluate the effects of XZK on the kidneys and investigate the related mechanisms in a rat model of hyperlipidemia. Thus, the effect on inflammatory transcription factors and kidney damage was investigated with *in vitro* and *in vivo* experiments on hyperlipidemic rats following XZK treatment. The results revealed that the plasma levels of total cholesterol (TC), triglycerides (TG) and low-density lipoprotein-cholesterol (LDL-C) were significantly decreased, while the levels of high-density lipoprotein-cholesterol (HDL-C) were significantly upregulated in the XZK treatment group, as compared with those in the hyperlipidemia group (P<0.05). In addition, the results demonstrated that XZK was able to repair the kidney damage caused by hyperlipidemia. Furthermore, the expression levels of the inflammatory transcription factors, tumor necrosis factor-α and interleukin-6, were shown to be reduced in the XZK group when compared with the hyperlipidemia group. In summary, XZK reduces kidney injury, downregulates the levels of TG, TC and LDL-C, as well as the expression levels of inflammatory transcription factors, and upregulates HDL-C. These results further the understanding of the molecular pathogenic mechanisms underlying hyperlipidemia and aid the development of XZK as an effective therapeutic agent for hyperlipidemia.

## Introduction

Hyperlipidemia is a lipid metabolism disorder that causes abnormally elevated levels of cholesterol, triglycerides (TG) and lipoproteins in the blood (1). Hyperlipidemia is widely accepted to be a key risk factor for atherosclerosis, coronary heart disease (CHD) and peripheral vascular disease (2,3). Decreasing the lipid levels has been shown to reduce the risk of coronary artery disease and aid in preventing additional coronary events (4). In addition, hyperlipidemia may result in solid organ injury, including damage to the liver and kidneys (5) Statins are considered to be the first-line therapy in the reduction of lipid levels, and large clinical outcome trials have consistently demonstrated their efficacy in low-density lipoprotein-cholesterol (LDL-C) reduction and the prevention of cardiovascular events (6). However, the application of statins is restricted by the adverse side-effects on liver function and creatine kinase activity, particularly in older patients, those with multiple comorbid diseases and those treated with high-dose statins or a combination lipid-lowering therapy. Thus, research into an effective, safe, alternative therapy for CHD patients with the added complication of hyperlipidemia is of great clinical significance.

Xuezhikang (XZK), an extract from the red yeast rice *Monascus purpureus*, has been used as a traditional Chinese medicine for patients with cardiovascular diseases for over 2,000 years (7). XZK contains 13 types of natural statins, as well as unsaturated fatty acids, flavonoids, ergosterol, amino acids, alkaloids, trace elements of plant sterols and a number of other biologically active substances (8); thus, XZK is regarded as a natural polypill. A number of studies have demonstrated that fermented red yeast rice not only moderately lowers cholesterol levels, but has the additional advantage of causing fewer adverse effects compared with other statin drugs (9–11). In addition, a previous study demonstrated that XZK significantly reduces the recurrence of coronary events and the occurrence of new cardiovascular events and mortalities, in addition to improving lipoprotein regulation and being a safe and well-tolerated drug (12). Combining XZK with other therapies in the treatment of fatty liver has been reported to relieve clinical syndromes and improve liver function (5). However, the effects of XZK treatment on the liver in patients with hyperlipidemia have not yet been fully identified.

Therefore, the aim of the present study was to investigate the effects of XZK treatment on the plasma levels of total cholesterol (TC), TG and LDL-C. In addition, the effects of XZK treatment on kidney function and the related molecular mechanisms were evaluated in a rat model of hyperlipidemia.

## Materials and methods

### Materials

XZK capsules (0.6 g each) were obtained from Beijing Peking University WBL Biotech Co., Ltd. (Beijing, China). Each capsule contained a combination of lovastatin, also known as monoclonin K (2.5–3.2 mg/capsule), and small quantities of lovastatin hydroxyl acid, unsaturated fatty acids (primarily linoleic acid, oleic acid, palmitic acid and stearic acid), essential amino acids, ergosterol and a number of other components. The bioavailability of lovastatin contained within XZK was 169% compared with that of purified lovastatin (13).

### Animal treatment

A total of 30 male Sprague-Dawley rats, weighing 180–200 g, were purchased from the Laboratory Animal Institute of Jilin University (Changchun, China). Animals were housed in an environmentally controlled breeding room, under the institutional guidelines of the Experimental Animals of Jilin University for the humane treatment of laboratory animals.

Animals were randomly divided into three groups, with ten rats in each group. Rats in group I (control group) were fed a normal diet, while rats in the other two groups were fed a high cholesterol diet with vitamin D3 (Sigma-Aldrich, St Louis, MO, USA) added to normal chow. After six weeks, blood was collected from the tails of the rats and the lipid and lipoprotein levels were determined. To ensure that all the rats in the high-fat diet-fed groups were hyperlipidemic, the animals were subsequently treated with the following pharmacological agents. Group III rats (XZK group) were fed a high cholesterol diet plus vitamin D3 and XZK (300 mg/kg, p.o.) daily for 42 days, while rats in Group II (hyperlipidemic group) were fed a high cholesterol diet plus vitamin D3 and administered saline daily for 42 days. The body weight and food intake of each rat were recorded during the experimental period. Animal surgery was performed under sodium pentobarbital anesthesia. The experimental protocols were approved by the Institutional Animal Ethics Committee of Jilin University.

### Blood samples and kidney tissues

The rats were monitored daily for general health and weighed individually at the beginning and end of the experiment. The daily feed intake and weight gain were recorded during the experimental period.

At the end of the experiment, all the rats were anesthetized with sodium pentobarbital (1.25 g/kg; Sigma-Aldrich) following overnight fasting, and decapitated. Blood samples were collected by cardiac puncture and were maintained at room temperature for coagulation. The serum was obtained by centrifugation at 3,000 × g at 4°C for 10 min, after which the samples were stored at −70°C for later use. Kidneys were excised, rinsed in ice-cold saline, blotted dry on filter paper and weighed. Aliquots of the kidney were snap-frozen in liquid nitrogen for 24 h and stored at −70°C prior to use. A portion of each kidney was fixed in 10% formalin for histological analysis.

### Measurement of lipid and lipoprotein levels

Serum levels of TC, TG, high-density lipoprotein-cholesterol (HDL-C) and LDL-C were measured enzymatically using commercially available kits (Randox Laboratories, San Francisco, CA, USA), according to the manufacturer’s instructions.

### Determination of renal antioxidant activity and malondialdehyde (MDA) content

Catalase (CAT) activity was measured as the reduction in H_2_O_2_ concentration by recording the absorbance at a wavelength of 240 nm (14). Glutathione peroxidase (GSH-px) activity was assayed as previously described (15), while the levels of superoxide dismutase (SOD) and MDA were determined using diagnostic kits (Jiangchen Science & Technology Co., Ltd., Nanjing, China) with a UV-visible spectrophotometer (Shimadzu Corporation, Kyoto, Japan), according to the manufacturer’s instructions.

### Assay for renal function parameters

Blood samples were obtained from the rats for the measurement of glucose, serum creatinine (Scr), blood urea nitrogen (BUN), uric acid (UA), urine creatinine (Ucr) and albuminuria. The serum levels were detected using diagnostic kits (Jiangchen Science & Technology Co., Ltd.). The kidney index was calculated as follows: 1,000 - kidney weight/body weight. Creatinine clearance (Ccr) was calculated according to the following formula (16): Ccr (ml/kg/min) = urinary creatinine (μM) × urinary volume (ml/kg/min)/Scr (μM).

### Assays for renal histology

Renal tissue samples were fixed in 10% (v/v) neutral buffered formalin and embedded in paraffin wax. Paraffinized sections were cut into 5-μm sections, deparaffinized with xylene and dehydrated with ethanol. The sections were stained with hematoxylin and eosin (HE) for histopathological analysis with a photomicroscope (Olympus, Tokyo, Japan). The operative procedures complied with the standard protocols and the examination of the slides was performed by a pathologist blinded to the experimental profile.

### Measurement of serum tumor necrosis factor (TNF)-α and interleukin (IL)-6

A sandwich enzyme-linked immunosorbent assay (ELISA) was used to determine the serum concentrations of TNF-α in all the rats after six weeks of XZK treatment, using a rat TNF-α DuoSet ELISA development kit (eBioscience, San Diego, CA, USA). Briefly, 96-well ELISA plates were coated with a monoclonal mouse anti-human TNF-α antibody and incubated overnight at 4°C, following which the plates were washed three times with phosphate-buffered saline containing 0.05% Tween 20 (PBST). The plates were incubated with blocking solution for 1 h at room temperature and the test samples and recombinant TNF-α standards were added. Subsequently, the plates were incubated at room temperature for a further 2 h, following which they were washed five times with PBST. Biotin-conjugated anti-human TNF-α antibodies were added, and the plates were incubated at room temperature for 2 h and washed with PBST. Avidin-horseradish peroxidase (HRP) was added and the reaction was allowed to proceed at room temperature for 30 min. The plates were washed five times with PBST, and 3,3′,5,5′-tetramethylbenzidine (Sigma-Aldrich, Sydney, Australia) was added to develop the reaction, which was stopped by H_2_SO_4_ (2N). The optical density was measured at a wavelength of 450 nm with a VERSAmax automated microplate reader (Molecular Devices, Inc., Sunnyvale, CA, USA). A standard curve was constructed by plotting the optical density values against the log of the TNF-α concentration.

The aforementioned ELISA protocol was similarly completed using the rat IL-6 DuoSet ELISA development kit (eBioscience) to determine the sera levels of IL-6.

### Quantitative reverse transcription polymerase chain reaction (RT-qPCR)

All glassware was treated with diethyl-pyrocarbonate to inhibit RNases. Total RNA was extracted from the kidneys of 30 rats (n=10 per group) using TRIzol^®^, according to the manufacturer’s instructions (Invitrogen Life Technologies, Carlsbad, CA, USA). Reverse transcription was performed using a *Taq*Man Reverse Transcription kit (Applied Biosystems, Foster City, CA, USA) in a total reaction volume of 100 μl with 2 μg total RNA. RT-qPCR was performed in a total reaction volume of 25 μl using *Taq*Man fast mix (Applied Biosystems). Gene expression was normalized against the expression level of β-actin. The specific primers used were as follows: TNF-α forward, 5′-CGAGTCTGGGCAGGTCTACTTT-3′ and reverse, 5′-AAGCTGTAGGCCCCAGTGAGTT-3′; IL-6 forward, 5′-ATGCCTGACCTCAACTCCACT-3′ and reverse, 5′-GAGCAGCCCCAGGGAGAA-3′; β-actin forward, 5′-GATCATTGCTCCTCCTGAGC-3′ and reverse, 5′-ACTCCTGCTTGCTGATCCAC-3′. The PCR thermal cycling conditions were as follows: Initial denaturation at 95°C for 3 min, followed by 30 cycles of denaturation for 30 sec at 95°C, annealing for 40 sec at 58°C and extension for 30 sec at 72°C, with a final extension at 72°C for 10 min. The expression levels of the target genes were normalized against that of β-actin in the cDNA samples. The experiments were performed in triplicate.

### Western blot analysis

All kidney specimens were weighed, lysed, homogenized and centrifuged at 14,000 × g for 15 min at 4°C. The kidney protein content of the supernatant was assayed using Bradford reagent (Sigma-Aldrich, Steinheim, Germany). After ensuring the linearity of the band density, samples (10 μg total protein) were applied to 10–15% polyacrylamide gels, where the proteins were separated using standard SDS-PAGE protocols and transferred to polyvinylidene difluoride membranes (Bio-Rad Laboratories, Inc., Hercules, CA, USA). Subsequently, the membranes were blocked with 5% bovine serum albumin (BSA) and incubated at room temperature for 2 h. This was followed by a second 2-h incubation in 5% BSA containing a rabbit anti-mouse TNF-α antibody (1:1,000, Santa Cruz Biotechnology, Inc., Santa Cruz, CA, USA) and a rabbit anti-mouse IL-6 antibody (1:500; Abcam, Cambridge, UK). Following three washes with PBS, the membranes were incubated with a goat anti-mouse secondary antibody conjugated to HRP (1:1,000; Wuhan Boster Biological Technology, Ltd., Wuhan, China) for 1 h at room temperature. Visualized bands were detected using an enhanced chemiluminescence reagent (Amersham Pharmacia Biotech, Little Chalfont, UK), and densitometric analysis of the protein bands was performed using Bio-Rad Quantity One software (Bio-Rad Laboratories, Inc.). The TNF-α and IL-6 protein content was expressed relative to β-actin in arbitrary units.

### Statistical analysis

All data are expressed as the mean ± standard deviation. Statistical comparisons of more than two groups were performed using one-way analysis of variance, followed by Dunnett’s post-hoc test. SPSS 16.0 for Windows (SPSS, Inc., Chicago, IL, USA) and Prism 5.0d (GraphPad Software, Inc., San Diego, CA, USA) software were used for statistical analyses. P<0.05 was considered to indicate a statistically significant difference.

## Results

### Food intake and body weight

**Figure 1:**
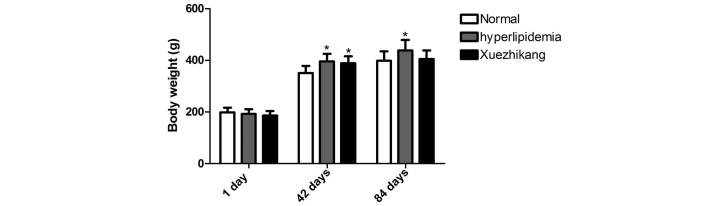
Body weight of the rats in the different experimental groups. Xuezhikang was administered on day 43 and the rats were sacrificed on day 85. Data are expressed as the mean ± standard deviation (n=10 per group). ^*^P<0.05, vs. control group.

In the course of the experiment, the daily food intake of the XZK and hyperlipidemic rats was virtually identical, and less than that of the rats fed a normal diet. After 42 days, the hyperlipidemic group had a significantly increased body weight compared with the rats fed a normal diet. In addition, after 84 days, the average body weight of the rats fed the high-fat diet was significantly greater than that of the rats fed a normal diet (P<0.01), indicating that the model was successful at inducing hyperlipidemia. Administration of XZK reduced the weight gain (P<0.01) compared with the rats fed the high-fat diet alone (Fig. 1).

### Serum lipid and lipoprotein parameters

**Figure 2:**
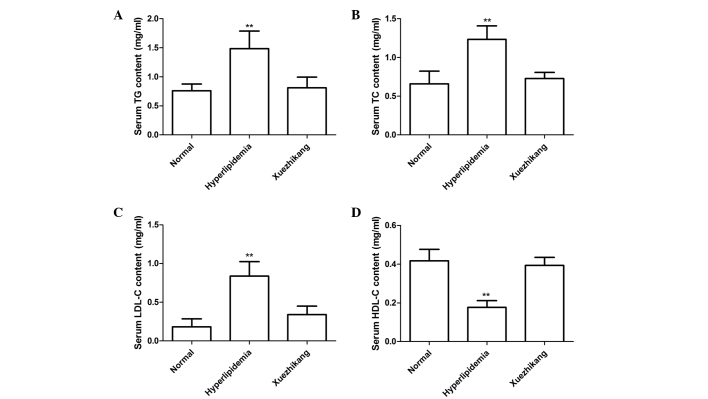
Serum lipid profiles of (A) TG, (B) TC, (C) LDL-C and (D) HDL-C in the different experimental groups.^**^P<0.01, vs*.* control group. TG, triglyceride; TC, total cholesterol; LDL-C, low-density lipoprotein-cholesterol; HDL-C, high-density lipoprotein-cholesterol.

Compared with the rats fed the normal diet, rats fed the high-fat diet had significantly increased serum levels of TC (P<0.001) and TG (P<0.001). Administration of XZK (300 mg/kg) significantly reduced the serum levels of TC and TG compared with the rats fed the high-fat diet alone (P<0.001; Fig. 2A and B). In addition, following 12 weeks on the high-fat diet, the serum levels of LDL-C significantly increased (P<0.001), while the serum levels of HDL-C decreased (P<0.01), as compared with the control group. Administration of XZK (300 mg/kg) markedly reduced the serum levels of LDL-C (P<0.001) and increased the serum levels of HDL-C (P>0.05) compared with the rats fed the high-fat diet alone (Fig. 2C and D).

### Effects of XZK on the renal antioxidant status

**Figure 3:**
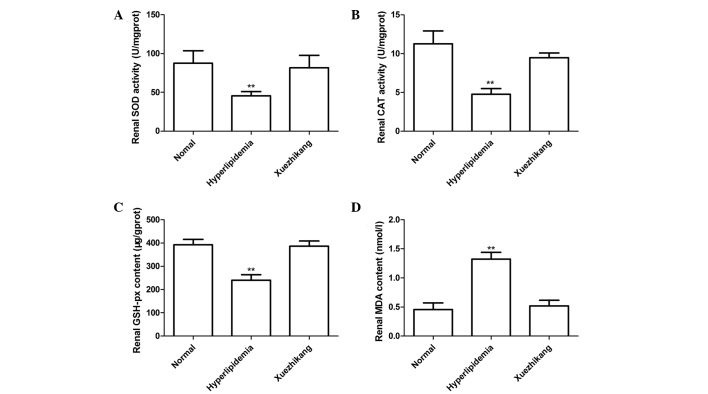
Oxidative stress was assessed by analyzing the (A) renal SOD activity, (B) renal CAT activity, (C) renal GSH-px content and (D) renal MDA content in the different experimental groups. Statistical significance was determined by one-way analysis of variance with Dunnett’s post-hoc test. ^**^P<0.01, vs. control group. SOD, superoxide dismutase; CAT, catalase; GSH-px, glutathione peroxidase; MDA, malondialdehyde.

Levels of renal SOD and CAT activity, and MDA and GSH-px for all the groups are presented in Fig. 3. The high-fat diet was shown to reduce the levels of SOD and CAT activity and the renal content of GSH-px, but increase the level of MDA (P<0.01; Fig. 3) when compared with the rats fed the normal diet. Daily administration of XZK (300 mg/kg) significantly increased the activity of SOD and CAT, as well as the levels of GSH-px, while reducing the levels of renal MDA (P<0.01; Fig. 3) when compared with the rats fed the high-fat diet alone.

### Effects of XZK on renal function

As shown in Table I, renal function was reflected by the levels of BUN, Scr, UA, Ccr and albuminuria. In the rats fed the high-fat diet, the levels of BUN, Scr, UA, Ccr and albuminuria were significantly higher compared with those in the rats fed a normal diet (P<0.01). However, administration of XZK significantly reversed these changes, with the levels of BUN, Scr, UA, Ccr and albuminuria reduced to near normal values (P<0.05).

### Histological evaluation

**Figure 4:**
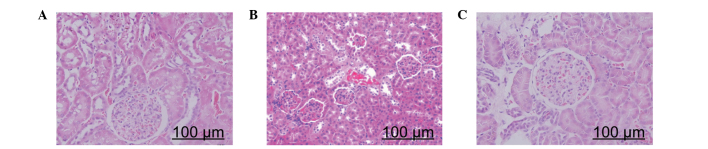
Photomicrographs of kidney samples stained with hematoxylin and eosin from the (A) normal, (B) hyperlipidemia and (C) Xuezhikang-treated groups. The hyperlipidemia tissue shows lymphocyte infiltration and vascular congestion, while the Xuezhikang-treated hyperlipidemia tissue shows significantly attenuated kidney damage.

Photomicrographs of kidney samples stained with HE are presented in Fig. 4. Kidney samples from the rats fed the normal diet for 12 weeks appeared normal (Fig. 4A). However, pathological lesions were observed in the kidney samples of rats fed the high-fat diet, which included epithelial cell necrosis of the proximal tubules, leucocyte infiltration, vascular congestion and tubular dilatation (Fig. 4B). Notably, the kidney injury was markedly reduced following the administration of XZK (Fig. 4C).

### Serum levels of TNF-α and IL-6

**Figure 5:**
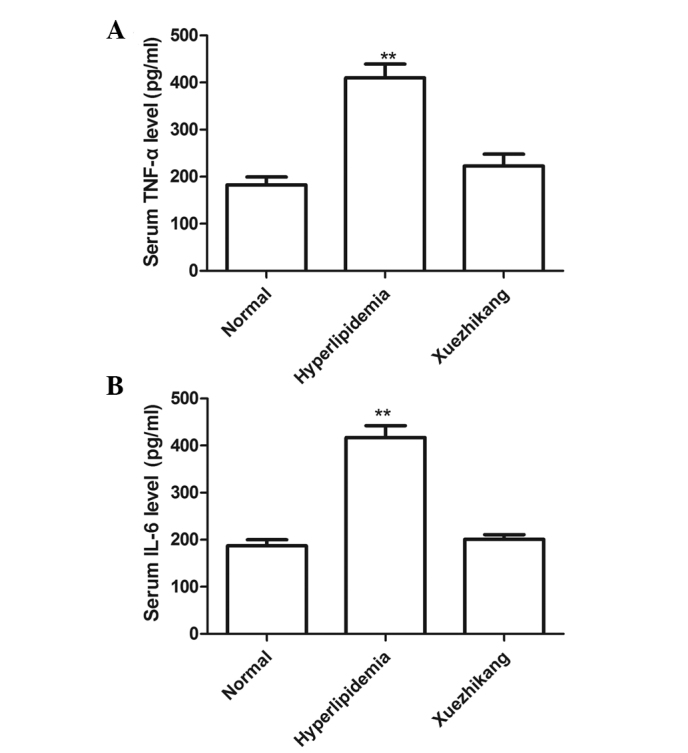
Serum expression levels of (A) TNF-α and (B) IL-6 in the experimental groups. ^**^P<0.01, vs. control group. TNF, tumor necrosis factor; IL, interleukin.

To measure the serum expression levels of TNF-α and IL-6 in all the groups, sandwich ELISAs were performed. As shown in Fig. 5, the serum levels of TNF-α and IL-6 in the high-fat diet group were higher than those of the rats fed a normal diet (P<0.01; Fig. 5A and B). The serum levels of TNF-α and IL-6 were reduced in the XZK treatment group when compared with those of the high-fat diet group, and no statistically significant difference was observed between the XZK treatment group and the normal diet group.

### TNF-α and IL-6 expression levels in the kidney tissue

**Figure 6:**
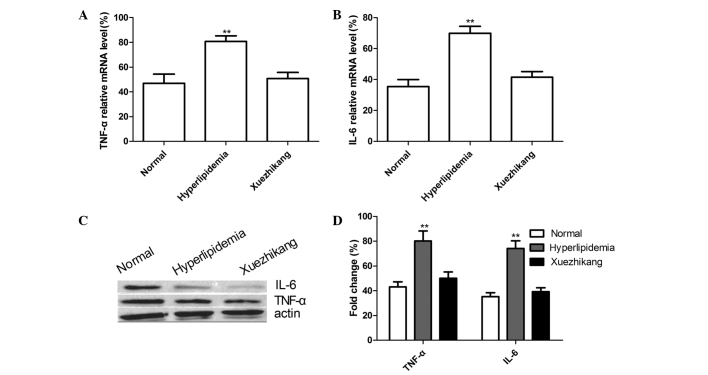
Renal expression levels of TNF-α and IL-6 in the different experimental groups. Relative mRNA expression of (A) TNF-α (fold-change of protein expression) and (B) IL-6 (fold-change of protein expression). (C) Western blot and (D) quantitative analyses (fold-change of protein expression) showing the protein expression levels of TNF-α and IL-6. ^**^P<0.01, vs. control group. TNF, tumor necrosis factor; IL, interleukin.

To measure the mRNA and protein expression levels of TNF-α and IL-6 in the kidney tissues of all the groups, RT-qPCR and western blot analysis were performed, respectively. As shown in Fig. 6A and B, the mRNA expression levels of TNF-α and IL-6 in the high-fat diet group were significantly higher compared with the normal diet group (P<0.01). When compared with the high-fat diet group, the TNF-α and IL-6 mRNA expression levels in the XZK treatment group were significantly reduced (P<0.05). Additionally, the protein expression levels of TNF-α and IL-6 were increased in the high-fat diet group when compared with those in the normal diet group (P<0.01), and the administration of XZK markedly reduced the TNF-α and IL-6 protein expression levels when compared with the high-fat diet group (P<0.05; Fig. 6C and D).

## Discussion

Epidemiological and clinical evidence supports the hypothesis that hyperlipidemia contributes to the formation and progression of atherosclerosis, which is an important factor in the pathogenesis of CHD (17). Reducing lipid levels may slow the progression of the disease (18). The Physician’s Health Study investigated the probability of developing renal dysfunction in 4,483 healthy male physicians, and found that the relative risk for elevated creatinine levels was directly associated with baseline blood lipid levels, and that dyslipidemia may cause chronic renal disease (19). In addition, the Helsinki Heart Study described an association between cholesterol levels and progressive kidney disease in 2,702 middle-aged dyslipidemic males (20). A number of studies on patients with hyperlipidemia have observed an association between the baseline levels of serum cholesterol and the probability of nephropathy progression (21–24).

The results of the present study revealed that the levels of BUN, Scr, UA, Ccr and albuminuria in rats fed a high-fat diet were significantly higher compared with those in rats fed a normal diet. In addition, the high-fat diet was demonstrated to cause kidney damage, which confirmed that hyperlipidemia may result in a loss of kidney function and kidney injury. The results also revealed that the hyperlipidemic rats developed higher serum levels of TG, TC and LDL-C, as well as a reduced concentration of HDL-C, which is consistent with the results of previous studies (25,26).

Statins are inhibitors of 3-hydroxyl-3-methylglutaryl Coenzyme A reductase, and have been shown to be the most efficacious therapy for hyperlipidemia. Statins not only reduce the plasma concentration of LDL-C, but also reduce cardiovascular morbidity and mortality in patients with dyslipidemia (27). However, for the majority of patients, statins alone are insufficient to achieve current guideline-recommended LDL-C goals. In addition, the safety of statins at high doses is a concern, in older patients where they may affect liver function (28). A recent study showed that statins delay the progression of kidney disease in a variety of animal experimental models (29). Thus, research into a safe yet effective therapeutic agent for use in the place of statins for the treatment of dyslipidemia and CHD is of great clinical significance. XZK, an extract of red yeast rice, has been shown to significantly reduce serum levels of TC, TG and LDL-C and increase serum levels of HDL-C in hyperlipidemic rat models and patients; the lipid modification effects of XZK appear to be similar to those of pravastatin, simvastatin, lovastatin and atorvastatin (30). In addition, XZK has been demonstrated to be a safe and effective treatment for the secondary prevention of CHD in older individuals (5).

The results of the current study demonstrated that the administration of XZK (300 mg/kg) significantly reduced the levels of TC, TG and LDL-C, and increased the levels of HDL-C (P<0.001) when compared with the rats fed a high-fat diet alone, which is in agreement with the results of previous studies (5,9–11). In addition, the results demonstrated that administration of XZK (300 mg/kg) restored the damaged kidney tissue and significantly reduced the levels of SOD, BUN, Scr, UA Ccr and albuminuria, while increasing the levels of MDA, CAT and GSH-px, as compared with the rats fed a high-fat diet. These results are consistent with a previous study (16), indicating that XZK is a safe and effective therapeutic agent that is able to reduce kidney injury caused by dyslipidemia.

Lipid deposition within arterial subintimal space is considered to be an important step in the formation of atherosclerotic plaques, preceding the increased expression of adhesion molecules on the endothelial cell surface and the subsequent leukocyte recruitment from the blood stream (31). Proinflammatory cytokines are hypothesized to have an important role in lipopolysaccharide-mediated endothelial damage, through the uptake of oxidized LDL via increased expression levels of macrophage scavenger receptors (32), and by regulating plaque stability (33), which may be important processes in the pathogenesis of atherosclerosis (34). However, the area in which the cytokines are produced is important. In the plasma, TNF-α and IL-6 may induce endothelial cell damage (35), whereas cytokines produced in the atherosclerotic plaque stimulate cell proliferation and the migration of smooth muscle cells and macrophages (34), reducing the stability of the plaque (33). Thus, the downregulation of TNF-α and IL-6 is necessary in the treatment of cardiovascular disease. In the present study, XZK was shown to reduce the serum expression levels of TNF-α and IL-6 in the hyperlipidemia rat model. In addition, the levels of TNF-α were higher in the hyperlipidemia group than in the healthy control rats, demonstrating that hyperlipidemia causes the upregulation of proinflammatory cytokines and leads to liver tissue damage. Normal expression levels of TNF-α and IL-6 in the blood of healthy individuals aid the prevention of infection and enhance immune function; however, overexpression may result in injury to tissues (36). In the present study, the renal expression levels of TNF-α and IL-6 were increased in the hyperlipidemia group when compared with the normal control group, and XZK treatment was shown to reduce TNF-α and IL-6 expression compared with the hyperlipidemia group, indicating that TNF-α and IL-6 may be involved in kidney damage, and XZK may function by reducing the expression levels of TNF-α and IL-6.

In conclusion, the present study demonstrated that the administration of XZK (300 mg/kg) reduces kidney injury caused by hyperlipidemia, downregulates the levels of TG, TC and LDL-C, as well as the expression levels of inflammatory transcription factors, and upregulates HDL-C. These results are likely to contribute towards improving the understanding of the molecular pathogenesis and mechanisms underlying hyperlipidemia, and may aid the development of XZK as an effective therapeutic agent for hyperlipidemia.

## Figures and Tables

**Table I:** Renal functional parameters of the normal, hyperlipidemia and Xuezhikang groups after six weeks.

Renal parameters	Control	Hyperlipidemia	Xuezhikang
Albuminuria (μg/24 h)	21.12±6.34	110.89±12.14^b^	32.18±7.89
BUN (mM)	8.54±0.82	12.11±0.81^b^	8.64±0.94
Scr (μM)	97.24±6.82	148.88±8.54^b^	103.55±9.11
Ucr (μM)	487.34±26.44	1245±100.35^b^	589.89±45.23^a^
UA (μM)	58.87±6.94	83.23±10.45^b^	56.77±8.45
Ccr (ml/kg/min)	3.64±0.26	6.43±0.42^b^	3.67±0.27

a P<0.05 and

b P<0.01, vs. control.

BUN, blood urea nitrogen; Scr, serum creatinine; Ucr, urine creatinine; UA, uric acid; Ccr, creatinine clearance.
